# Phenotypic screening in the 21st century

**DOI:** 10.3389/fphar.2014.00264

**Published:** 2014-12-01

**Authors:** Birgit T. Priest, Gul Erdemli

**Affiliations:** ^1^Eli Lilly & Co.Indianapolis, IN, USA; ^2^NovartisCambridge, MA, USA

**Keywords:** drug discovery, systems biology, label-free detection, phenotypic screening, gene networks

Since the advent of molecular cloning, target based screening has become the norm in pharmaceutical drug discovery. A large number of potential drug targets have been cloned and functionally expressed, and enormous progress has been made in the development, miniaturization and automation of cell based assays on target molecules recombinantly expressed in mammalian cell lines. This approach has delivered many clinical candidates but relatively few new drugs. Target based screening is likely to provide very good drug candidates for monogenic diseases, and the following collection of manuscripts is not meant to discourage the use of target based approaches. However, most of the more prevalent human diseases are most likely multifactorial and require interaction with multiple targets to produce clinically meaningful efficacy. In addition, high potency, selective interaction with a single target may increases the risk of adverse events or be limited by redundancies and adaptive resistance. Here, target agnostic approaches using phenotypic assays may offer significant benefit. Making such approaches viable requires addressing a number of challenges. This e-book attempts to discuss some of these challenges and illustrate recent advances.

Prior to the 1980s, most drugs were discovered using phenotypic assays in live animals or isolated tissues (Swinney, 2013). Most of these drugs interact relatively weakly with a number of targets, and the complete profile of their molecular interactions is not well-known. Examples include most anti-convulsants, diuretics, and vasodilators. Screening *in vivo* typically returns compounds with acceptable ADME properties and access to the target *in vivo*; issues that often frustrate the present target based drug discovery programs.

Screening *in vivo* may be feasible, if a good starting point for Medicinal Chemistry and a clear strategy for differentiation exist. An example is the development of carbamazepine analogs with improved ADME properties (Landmark and Johannessen, 2008). In oncology, mouse xenograft models, based on a patient's tumor cells, have been used to select between drugs (Wu et al., 2012). However, many drug discovery efforts rely on screening to identify starting points for Medicinal Chemistry, and screening *in vivo* in most cases is not commercially or ethically viable. In this case, phenotypic screening relies on an appropriate tissue- or cell-based assay that can be miniaturized and used in combination with high throughput tools. Promising examples include anti-microbial assays, cell proliferation, platelet aggregation, and insulin release from pancreatic β-cells. For CNS diseases involving neuronal networks, selection of an appropriate substrate is especially difficult. The article by Bruni et al. discusses the potential for using zebrafish as a model system, enabling large scale behavior based screens for central nervous system disorders. However, as pointed out by Bruni et al. there are substantial differences in zebrafish and human biology and translation from zebrafish to human may be problematic. In order to limit the reliance on non-predictive animal models, screening strategies utilizing human pluripotent stem cells (hPSCs) are increasingly considered as resources for drug discovery. However, several hurdles need to be overcome before widespread implementation of hPSC-based assays. Viswanathan and colleagues reviewed the recent progress in the culture of hPSCs with emphasis on the importance of the environment surrounding these cells and high content analysis approaches for assay development.

The limited throughput of phenotypic assays compared to most target-based assays necessitates smaller libraries that are optimized with regard to biological and chemical diversity. Wassermann and colleagues discuss strategies for building appropriate, well-annotated compound libraries. Such libraries may also be used to identify pathways underlying the observed effects.

Another challenge involves the identification of a phenotypic end point associated with the disease of interest. In the early phases of drug discovery, phenotypic assays can be used to further our knowledge of the disease process and to identify those end points that are most likely to translate to the clinic. Furthermore, probability of success is greatly increased, if the end point measured can serve as a clinical biomarker. The perspective by Dr. Swinney analyzes the phenotypic end points used in the discovery of new molecular entities that have resulted from phenotypic drug discovery efforts.

An additional hurdle to translation is the large genetic diversity in the human population and the need to identify the right patient population for any given drug. Progress has been made in this regard in oncology. Ross et al. outline the approach taken for cancer and the potential application to other diseases. Similarly, the manuscript by Dr Schadt and colleagues points to the importance of gene networks for complex trait diseases and highlights the importance of understanding these networks in the appropriate biological context. In this regard, the integration of panomic data will be increasingly important.

Label-free technologies can offer advantages for phenotypic screening in that they do not involve assumptions about molecular mechanisms and pathways. The manuscript by Dr. Fang reviews the use of label-free technologies with a focus on Dynamic Mass Redistribution (DMR). Many label-free technologies offer the potential to be used as live cell kinetic assays. As discussed by Dawson and Carragher, this is especially important for studying drug combinations, since clinically successful combinations require compatible pharmacokinetics of the individual components. High content imaging is an attractive technology in this regard, especially since it is compatible with organotypic co-cultures. High content imaging can be combined with proteomics to identify pathways and biomarkers, and Dawson and Carragher describe some of the advances to increase throughput of proteomics.

We would like to thank the authors for their outstanding contributions and willingness to share their knowledge which made this Special Topic possible. All the manuscripts have been peer-reviewed and we are grateful to the expert reviewers for their valuable comments.

## Conflict of interest statement

The authors are employees of Eli Lilly & Co and Novartis, respectively. The authors declare that the research was conducted in the absence of any commercial or financial relationships that could be construed as a potential conflict of interest.
